# Impact of serum vitamin D on the response and prognosis in breast cancer patients treated with neoadjuvant chemotherapy

**DOI:** 10.1007/s12282-021-01292-3

**Published:** 2021-09-06

**Authors:** Eriko Tokunaga, Takanobu Masuda, Hideki Ijichi, Wakako Tajiri, Chinami Koga, Yumiko Koi, Yoshiaki Nakamura, Shinji Ohno, Kenichi Taguchi, Masahiro Okamoto

**Affiliations:** 1 Department of Breast Oncology, National Hospital Organization Kyushu Cancer Center, 3-1-1 Notame, Minami-ku, Fukuoka, 811-1395 Japan; 2 Departments of Pathology, National Hospital Organization Kyushu Cancer Center, 3-1-1 Notame, Minami-ku, Fukuoka, 811-1395 Japan; 3 Breast Cancer Center, Cancer Institute Hospital, 3-8-31 Ariake, Koutou-ku, Tokyo, 135-8550 Japan

**Keywords:** Vitamin D, Breast cancer, Neoadjuvant chemotherapy, Pathological complete response, Prognosis

## Abstract

**Background:**

Several studies have recently reported that the relationships between serum vitamin D and the prognosis or the pathological response to neoadjuvant chemotherapy (NAC) in breast cancer. However, there are no data regarding the clinical impacts of the vitamin D in Japanese breast cancer patients so far.

**Patients and methods:**

In the present study, a total of 250 patients with clinical Stage I–III primary breast cancer who were treated with NAC and subsequently underwent definitive surgery were included. Serum 25-hydroxvitamin D (25(OH)D) levels were evaluated using blood samples obtained before NAC.

**Results:**

The serum 25(OH)D was positively associated with age, and the serum 25(OH)D was significantly higher in postmenopausal women than that in pre/peri-menopausal women. Serum 25(OH)D level was not associated with the achievement of pathological complete response (pCR) in this cohort. The low 25(OH)D levels were significantly associated with shorter time to distant recurrence (TTDR). According to the univariate analysis, high clinical stage before NAC (cStage III) and low serum 25(OH)D level were significantly associated with the shorter TTDR, and pCR was significantly associated with the longer TTDR. According to a multivariate analysis, low serum 25(OH)D level were independent poor prognostic factors for TTDR.

**Conclusions:**

The low 25(OH)D levels were significantly associated with poorer prognosis in Japanese women with operable breast cancer patients treated with NAC.

## Introduction

Vitamin D is a fat-soluble secosteroid with well-established effects on calcium homeostasis, and it is the precursor to the potent steroid hormone calcitriol [1, 2]. Deficiency of vitamin D is associated with increased risks of morbidity and mortality in various diseases including cardiovascular, malignant, and autoimmune diseases [2–5]. Vitamin D regulates numerous cellular pathways such as inhibition of cellular proliferation, inflammation, invasion, metastasis and angiogenesis, and also promotion of apoptosis and differentiation [2]. One of those mechanisms is the intervention on the cell cycle via kinases such as cyclins, cyclin-dependent kinases (CDKs) and CDK physiological modulators [6]. Previous studies have revealed that the vitamin D deficiency was associated with poor outcomes in breast cancer [7–9], although contradictory results have also been reported [10, 11].

25-hydroxvitamin D (25(OH)D) is the circulating form of vitamin D that is measured in the blood and clinically used to establish and monitor the vitamin D status. One study showed that the serum 25(OH)D levels could predict cancer survival in a prospective cohort with measurements prior to and at the time of cancer diagnosis [12].

Neoadjuvant chemotherapy (NAC) has become a standard of care not only in locally advanced breast cancer, but in early breast cancer, especially for patients with large tumor size, lymph node metastasis, HER2 overexpression and triple negative breast cancer (TNBC) subtype. Pathological complete response (pCR) is associated with improved prognosis. This association appears stronger in the HER2+ /hormone receptor (HR)—or TNBC subtype [13, 14].

Recently, a statistically significant association between 25(OH)D level at baseline and pCR in patients with receiving NAC has been reported [15, 16]. However, other studies reported that there was no effect of serum 25(OH)D levels on the pathologic complete response (pCR) in neoadjuvant settings [17–19].

Recent reports shows that East Asian countries have high prevalence rates of Vit D deficiency [20, 21]. However, in Japan, the importance of the vitamin D on the incidence of cancer or the prognosis of the patients with malignancies has not been recognized so much. There are few studies on the association between serum vitamin D levels and the clinicopathological features and the prognosis of the Japanese women with breast cancer. In the present study, we retrospectively compared serum 25(OH)D level at baseline and the efficacy of NAC and the prognosis after surgery.

## Patients and methods

### Patient population

A total of 250 patients with clinical Stage I–III primary breast cancer who were treated with NAC and subsequently underwent definitive surgery of the breast and axilla in the Department of Breast Oncology, National Hospital Organization Kyushu Cancer Center, between 2009 and 2019 were included in this study. Chemotherapy was administered according to the standard regimens of anthracyclines and/or taxanes with or without trastuzumab for HER2-positive (HER2+) breast cancer. For all these patients, serum obtained just before starting NAC was available. The clinical data were obtained from the patients’ medical records. The anatomical AJCC/UICC TNM classification and stage groupings were used. This study was approved by the institutional review board of our hospital, and was in accordance with the Declaration of Helsinki. The serum was the surplus sample from the usual blood test. We obtained the written informed general consent from an individual patient before the start of the treatment and the sample acquisition. Our IRB waived the requirement for obtaining another written informed consent when we use the surplus samples.

### Pathological examinations

All pathological examinations were performed by the experienced pathologists in our hospital. The expression of estrogen receptor (ER) and progesterone receptor (PgR) was regarded as positive if the nuclear expression was ≥ 1%. The HER2 status was evaluated according to the recommendation of ASCO/CAP [22]. The pCR was regarded as the total disappearance of invasive carcinoma cells, including lymph nodes, regardless of the presence of residual ductal carcinoma in situ. If ER and /or PgR were positive, it was defined HR-positive (HR+). The tumor subtypes were divided into the following four groups; HR+ /HER2−, HR+ /HER2+ , HR−/HER2+ and triple negative (TN; ER−, PgR− and HER2−).

### Serum vitamin D (25-hydroxyvitamin D; 25(OH)D) levels

Serum samples obtained before the first cycle of NAC were stored at − 30 °C until analysis. Serum 25(OH)D levels were analyzed by ELISA at SRL laboratories (Tokyo, Japan). Serum 25(OH)D levels were defined as ‘sufficient’ if serum 25(OH)D ≥ 20 ng/mL and ‘insufficient’ if serum 25(OH)D < 20 ng/mL [2, 19].

### Statistical analyses

The statistical analyses were performed using the JMP software package, version 14.0 (SAS Institute Inc., Cary, NC, USA). The associations between the clinicopathological characteristics were assessed using *χ*^2^ tests. The time to recurrence (TTR) was defined as the time from surgery to the first breast cancer event, including loco-regional recurrence, distant metastasis or a new cancer in the contralateral breast. The time to distant recurrence (TTDR) was defined as the time from the date of curative surgery to the detection of distant recurrence. The overall survival (OS) was defined as the time from the date of curative surgery to death. Survival curves were plotted using the Kaplan–Meier method, and the association between the survival and each variable was determined by the log-rank test. For multivariate analysis of the survival data, Cox proportional hazards model was used. Differences were considered to be significant at *P* < 0.05.

## Results

### Patients’ characteristics

The clinicopathological characteristics of the patients included in this study were shown in Table 1. The median age was 59 years (range 28–75). Pre/peri-menopausal and postmenopausal women were 135 (54.0%) and 115 (46.0%), respectively. About 60% of the patients were at clinical stage II. The rate of ER, PgR and HER2-positivity was 69.6%, 48.0% and 39.2%, respectively. In terms of tumor subtypes, HR+ /HER2− were 111 (44.4%), HR+ /HER2+ 67 (26.8%), HR−/HER2+ 31 (12.4%) and TN 41 (16.4%), respectively. Among the 250 patients, pCR was achieved in 60 (24.0%) patients.

**Table 1:** Clinicopathological characteristics of the patients

Factors	*N* (%)
Age (years)	
Median (range)	59 (28–75)
Menopausal status	
Pre/perimenopause	135 (54.0)
Postmenopause	115 (46.0)
Clinical stage	
I	6 (2.4)
IIA	73 (29.2)
IIB	89 (35.6)
IIIA	21 (8.4)
IIIB	24 (9.6)
IIIC	37 (14.8)
Nuclear grade	
1, 2	185 (74.6)
3	63 (25.4)
ER	
Negative	76 (30.4)
Positive	174 (69.6)
PgR	
Negative	130 (52.0)
Positive	120 (48.0)
HER2	
Negative	152 (60.8)
Positive	98 (39.2)
Subtype	
HR+/HER2−	111 (44.4)
HR+/HER2+	67 (26.8)
HR−/HER2+	31 (12.4)
TN	41 (16.4)
Pathological efficacy	
pCR	60 (24.0)
Non-pCR	190 (76.0)
25(OH)D	
Insufficient (< 20 ng/mL)	241 (96.4)
Sufficient (≥ 20 ng/mL)	9 (3.6)

ER: estrogen receptor, PgR: progesterone receptor, pCR: pathological complete response, HR: hormone receptor, TN: triple-negative, 25(OH)D: 25-hydroxyvitamin D

### Relationships between the serum 25(OH)D level and the clinicopathological characteristics

**Fig. 1:**
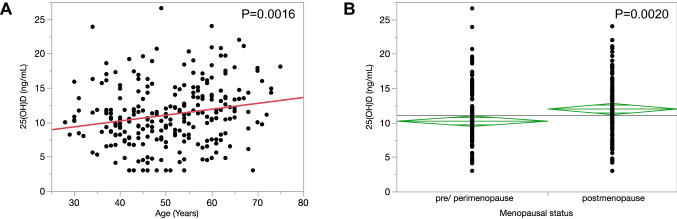
Relationship between age and menopausal status and the serum 25(OH)D level. **A** Relationship between age and the serum 25(OH)D level. **B** Relationship between menopausal status and the 25(OH)D level

The median 25(OH)D levels were 10.7 ng/mL (range 3.0–26.6 ng/mL). Surprisingly, serum 25(OH)D was sufficient in only nine patients (3.6%) in this cohort (Table 1). Serum 25(OH)D was positively associated with age (*P* = 0.0016), and serum 25(OH)D was significantly higher in postmenopausal women than that in pre/peri-menopausal women (*P* = 0.0020, Fig. 1). In the present study, serum 25(OH)D level was divided into two groups, low and high, using a dichotomous variable at the median value 10.7 ng/mL. Relationships between the serum 25(OH)D level and the clinicopathological characteristics were shown in Table 2. The median age of the high 25(OH)D group was significantly higher than that of the low 25(OH)D group (52.7 ± 0.93 year old vs. 48.5 ± 0.93, *P* = 0.0015), and the number of the postmenopausal women was significantly higher in the high 25(OH)D group (*P* = 0.0022). There were no significant associations between serum 25(OH)D level and the clinical stage, ER and PgR expressions, HER2 status and tumor subtypes.

**Table 2:** Relationships between the clinicopathological characteristics and the serum 25(OH)D level

Factors	25(OH)D low (*n* = 124)	25(OH)D high (*n* = 126)	*P* value
Age (years)			
Mean ± SE	48.5 ± 0.93	52.7 ± 0.93	0.0015
Menopausal status			
Pre/perimenopause	79 (63.7)	56 (44.4)	0.0022
Postmenopause	45 (36.3)	70 (55.6)	
Clinical stage			
I	2 (1.6)	4 (3.2)	0.147
IIA	35 (28.3)	38 (30.2)	
IIB	40 (32.3)	49 (38.9)	
IIIA	10 (8.1)	11 (8.7)	
IIIB	11 (8.9)	13 (10.3)	
IIIC	26 (21.0)	11 (8.7)	
Nuclear grade			
1, 2	85 (69.7)	100 (79.4)	0.0791
3	37 (30.3)	26 (20.6)	
ER			
Negative	36 (29.0)	40 (31.8)	0.6409
Positive	88 (71.0)	86 (68.2)	
PgR			
Negative	64 (51.6)	66 (52.4)	0.9033
Positive	60 (48.4)	60 (47.6)	
HER2			
Negative	75 (60.5)	77 (61.1)	0.9191
Positive	49 (39.5)	49 (38.9)	
Subtypes			
HR+/HER2−	55 (44.4)	56 (44.4)	0.9313
HR+/HER2+	35 (28.2)	32 (25.4)	
HR−/HER2+	14 (11.3)	17 813.9)	
TN	20 (16.1)	21 (16.7)	

*SE* standard error, *ER* estrogen receptor, *PgR* progesterone receptor, *HR* hormone receptor, *TN* triple-negative, *25(OH)D* 25-hydroxyvitamin D

### Relationships between serum 25(OH)D level and the efficacy of NAC

Then, the relationships between the clinicopathological factors and the efficacy of NAC were analyzed (Table 3). The low clinical stage (I and II), PgR negativity (*P* < 0.0001) and HER2 positivity (*P* < 0.0001) were significantly associated with the higher achievement of pCR. The HR+ /HER2− subtype was associated with the lower achievement of pCR (*P* < 0.0001). However, there was no significant association between the serum 25(OH)D level and the pCR.

**Table 3:** Relationships between the clinicopathological factors and pCR

	Non-pCR (*n* = 190)	pCR (*n* = 60)	*P* value
Menopausal status			
Pre/peri	104 (54.7)	31 (51.7)	0.6776
Post	86 (45.3)	29 (48.3)	
Clinical stage			
I, II	119 (62.6)	49 (81.7)	0.0045
III	71 (37.8)	11 (18.3)	
ER			
Negative	52 (27.4)	24 (40.0)	0.0681
Positive	138 (72.6)	36 (60.0)	
PgR			
Negative	83 (43.7)	47 (78.3)	< 0.0001
Positive	107 (56.3)	13 (21.7)	
HER2			
Negative	131 (69.0)	21 (35.0)	< 0.0001
Positive	59 (31.0)	39 (65.0)	
Subtype			
HR+/HER2−	100 (52.6)	11 (18.3)	< 0.0001
HR+/HER2+	42 (22.1)	25 (41.7)	
HR−/HER2+	17 (8.9)	14 (23.3)	
TN	31 (16.3)	10 (16.7)	
25(OH)D			
Low	97 (51.0)	27 (45.0)	0.4133
High	93 (49.0)	33 (55.0)	

*ER* estrogen receptor, *PgR* progesterone receptor, *pCR* pathological complete response, *HR* hormone receptor, *TN* triple-negative, *25(OH)D* 25-hydroxyvitamin D

### Relationships between serum 25(OH)D level and the prognosis

**Fig. 2:**
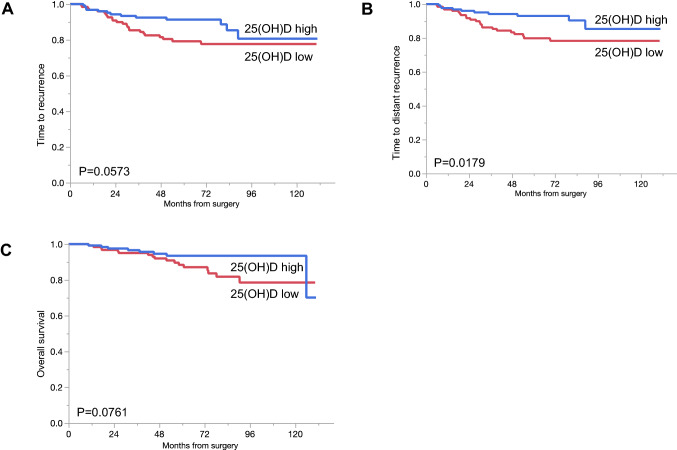
Relationship between the serum 25(OH)D level and the prognosis. **A** Time to recurrence, **B** time to distant recurrence, **C** overall survival. Red line: 25(OH)D low (*n* = 124), blue line: 25(OH)D high (*n* = 126)

Then, the relationships between serum 25(OH)D levels and the prognosis were evaluated. In spite of no associations with the efficacy of NAC, the prognosis of the patients with the low serum 25(OH)D level was associated with poorer prognosis. The TTDR was significantly shorter in the patients with low 25(OH)D level (*P* = 0.0179), and the TTR and OS was poorer in the patients with low 25(OH)D level, although not statistically significant (Fig. 2).

### Univariate and multivariate analyses of factors associated with TTDR

The clinicopathological factors possibly related to TTDR were analyzed by univariate and multivariate analyses (Table 4). According to the univariate analysis, high clinical stage before NAC (cStage III, *P* = 0.0165) and low serum 25(OH)D level (*P* = 0.0165) were significantly associated with the shorter TTDR, and pCR was significantly associated with the longer TTDR (*P* = 0.037). According to a multivariate analysis that included these factors, high clinical stage (cStage III) and low serum 25(OH)D level were independent poor prognostic factors for TTDR (*P* = 0.0445 and *P* = 0.0231, Table 4).

**Table 4:** Results of univariate and multivariate analyses for the TTDR

Factors	Parameters	Univariate analysis			Multivariate analysis		
		HR	95% CI	*P* value	HR	95% CI	*P*-value
Menopausal status	Pre/peri vs. post	0.94	0.47–1.90	0.8673			
Clinical stage before NAC	III vs. I, II	2.34	1.17–4.67	0.0165	2.05	1.01–4.10	0.0445
ER	Positive vs. negative	1.09	0.53–2.41	0.811			
PgR	Positive vs. negative	1.05	0.53–2.09	0.8842			
HER2	Positive vs. negative	0.601	0.26–1.25	0.1799			
Tumor subtypes	HER2, TN vs. HR+ /HER2−	0.57	0.28–1.12	0.1043			
Pathological efficacy	pCR vs. non-pCR	0.34	0.08–0.94	0.037	0.39	0.09–1.13	0.087
25(OH)D	Low vs. high	2.38	1.17–5.25	0.0165	2.28	1.12–5.03	0.0231

*HR* hazard ratio, *CI* confidence interval, *TTDR* time to distant recurrence, *pCR* pathological complete response, *ER* estrogen receptor, *PgR* progesterone receptor, *HR* hormone receptor, *TN* triple-negative, *25(OH)D* 25-hydroxyvitamin D

## Discussion

In the present study, we examined the relationships between the serum vitamin D (25(OH)D) levels and the pathological responses to NAC and the subsequent prognosis in Japanese women with operable breast cancer. There were no significant associations between the serum 25(OH)D levels and the pCR rate after NAC, however, the low serum 25(OH)D was significantly associated with the poor prognosis. As far as we know, this is the first study that investigated the association between the serum 25(OH)D levels and the efficacy of the NAC and the prognosis in the Japanese patients with breast cancer.

The association between vitamin D deficiency and the breast cancer risk and outcomes have been rigorously studied in Western countries, however, the data on the Asian women are lacking. For Japanese women, there is the only one case–control study using self-reported vitamin D intake, not serum 25(OD)D levels, which showed the significant inverse association with breast cancer [23]. In Korea, a large case–control study revealed that the deficient level of serum 25(OH)D was significantly associated with the breast cancer risk regardless of other breast cancer risk factors [24]. From these studies, vitamin D deficiency might be associated with the breast cancer risk in also Asian women.

In the Western countries, because there are lots of reports that showed the association between the vitamin D deficiency and the poor outcomes of breast cancer, the importance to intake sufficient vitamin D seems to have been recognized from an earlier time [7–9, 12]. In a study from Canada, women with early-stage breast cancer diagnosed prior to 2005 frequently had low levels of vitamin D, however, women diagnosed in 2008–2009, vitamin D levels were higher, probably because of the use of vitamin D supplements [9] Although there are few data of Asian breast cancer patients, a very recent study from Thailand showed that low serum 25(OH)D level was found to be independently associated with poor survival in breast cancer patients, regardless of age, lymph node status, stage or breast cancer subtype [25].

The associations between the serum 25(OH)D levels and the pathological responses to NAC were inconsistent among studies. In the reports by Chiba et al. and Viala et al. from the same group, a significant association between the serum 25(OH)D levels and the pathological responses to NAC (pCR) was recognized [15, 16]. On the other hand, in the patients enrolled in NEOZOTAC phase III trial or the study conducted in Korea, baseline and end of NAC 25(OH)D levels and the changes of the 25(OH)D levels were not related to the pathological responses [17, 19]. The rate of the patients with sufficient 25(OH) D levels are different among studies. More than 60% of the patients were at the sufficient level in the study by Chiba et al. and Viala et al. and NEOZOTAC trial. However, 25(OH) D were at sufficient level in only 16.8% in the study of Kim et al. In NEOZOTAC trial, only patients with HER2− breast cancer were included and other studies included the patients with both HER2− and HER2+ patients. In addition, pCR rate was different among studies. These factors might have influences on these inconsistencies.

In the present study, the low 25(OH)D was significantly associated with the poor prognosis and it was an independent poor prognostic factor by the multivariate analysis.

Surprisingly, serum 25(OH)D levels in most patients were insufficient (< 20 ng/mL) in our cohort. Serum 25(OH)D was positively associated with age, and serum 25(OH)D was significantly higher in postmenopausal women than that in pre/peri-menopausal women. Because the vitamin D deficiency is associated with the breast carcinogenesis, and the subjects of this study were all breast cancer patients, the vitamin D levels might have been lower than that of the healthy Japanese women. The low 25(OH)D levels in the breast cancer patients were also recognized in other studies of the Asian women [19, 24, 25]. In the Korean study of NAC, the median 25(OH)D levels were also significantly lower than that of the women in the Western countries, and the sufficient levels were more often observed in postmenopausal women, which are similar to our results [19]. Only two postmenopausal patients received an analogue of 1,25-dihydroxyvitamin D_3_ [1,25(OH)_2_D_3_]. The intake of these active vitamin D3 analog does not change the serum 25(OH)D level. Therefore, we think that the intake of active vitamin D3 analog had no effects of the result of this study.

As a consequence of indoor occupations and reduced exposure to sunlight, concerns for the vitamin D deficiency have been raised in developed countries [3, 4]. Also in Japan, females appear to be at higher risk of vitamin D deficiency because the younger people consume less fish than elders, they tend to avoid direct sunlight exposure to prevent skin-tanning, and they may be malnourished from maintaining a lean proportion [26–30]. We showed here the relationship between the low vitamin D and the poor prognosis in the Japanese women with primary breast cancer for the first time. We have not given the sufficient attention to the vitamin D in terms of the breast cancer risk and the prognosis in Japan. In addition, the analysis of 25(OH)D is not approved by the medical insurance in the ordinary practice of breast cancer in Japan. Therefore, the research regarding this theme might have not been done much in our country. We should pay more attention to vitamin D in regard to the cancer prevention and the prognosis.

The strength of the present study is that these data are form a single institution with high-quality follow-up and updated clinical data. However, these are several limitations associated with our study as well. All data are retrospective, and the duration of the follow-up was not sufficient. The number of the patients included in this study was small. In the present study, we included the only patients who underwent surgery after NAC. The cut-off value of the serum 25(OH)D was different from those in previous studies, most of which adopted 20 ng/mL as the cut off. To elucidate the association between serum vitamin D and the outcome of the breast cancer, the study using the large cohort which include the patients with or without NAC is necessary.

In conclusion, serum vitamin D level at the baseline was not associated with pCR rate after NAC, however, low serum vitamin D level was significantly associated with the poor prognosis in Japanese women. Vitamin D is important not only for the bone metabolism, but also in the cancer risk and prognosis. Therefore, we should pay more attention to the vitamin D intake. It is a great interest that the supplement of vitamin D could improve the prognosis of the breast cancer patients.
